# Nanoparticles for Effective Combination Therapy of Cancer

**Published:** 2016-10-30

**Authors:** Rahul Jadia, Cody Scandore, Prakash Rai

**Affiliations:** 1 Biomedical Engineering and Biotechnology Program, University of Massachusetts, University Avenue, Lowell, Massachusetts, US; 2 Department of Chemical Engineering, University of Massachusetts, University Avenue, Lowell, Massachusetts, US

**Keywords:** Nanomedicine, Oncology, Targeted Treatments, Chemotherapy, Photodynamic Therapy, Photothermal therapy, Drug Delivery, Biomedical Nanotechnology, Liposomes, Biotherapeutics

## Abstract

Cancer continues to remains a major healthcare problem across the world despite strong translational research efforts towards tackling the disease. Surgery, when possible, along with radiation and chemotherapy continue to remain the mainstay of cancer treatment. Novel targeted therapies or biologics and immunotherapies have recently been approved to improve treatment efficacies while reducing collateral damage to normal, non-cancerous tissues. Combination therapies have shown better results than individual monotherapies in the clinic but often the improvements in therapeutic indices remain marginal, at best. Several combinations treatments have been clinically approved for different types of cancer. Nanomedicine, the application of nanotechnology for medicine, has already made some positive impacts on the clinical care in this fight against cancer. Several nano-sized formulations of conventional chemotherapies have been clinically approved.

Nanotechnology provides a novel way to deliver combination therapies with spatiotemporal control over drug release. This review explores the recent advances in nanotechnology-mediated combination treatments against cancer. Multifunctional nanomedicines for mechanism-based combination therapies are likely to deliver the right drugs to the right place at the right time for optimal treatment responses with reduced morbidity. No nanomedicine that combines two or more drugs in a single platform has been approved for clinical use yet. This is because several challenges still remain in the development of nano-combinations including but not limited to - the optimal drug ratios in these nanomedicines, control over these drug ratios over multiple batches, large scale, reproducible manufacturing of these nanomedicines and cost of these nano-combinations among others. These challenges need to be addressed soon using a multidisciplinary approach with collaborations between academia, the pharmaceutical industry and the regulatory bodies involved to ensure that nano-combination therapy delivers on its promise of better treatment outcomes while severely reducing morbidity thus improving the quality of life in cancer patients.

## Introduction

Cancer represents a complex and heterogeneous family of diseases that has evaded full understanding because of its diverse attributes. While cancer survival rates have been steadily increasing since 1975, the American Cancer Society estimated 1,665,540 new cancer cases to occur in 2014 of which 585,720 cases were estimated to lead to death [1,2]. The current line of treatment includes surgery, chemotherapy, and radiation, or some combination of the three. The reason for increased difficulty in cancer treatment can be pinned on two major reasons: cancer is not a single disease and has variable components, and carcinogenesis is immensely complex. Cancer tissues are different due to random organization of the cells and various defects in the regulatory circuits and successive mutations in a cell line [3]. Therefore use of a single drug may prove to be impotent in inhibiting of these defects and mutations. The next generation of cancer therapy will combine conventional treatments to improve cytotoxic efficiency. The most recent FDA approved combination therapy is use of the lenvatinib capsule in combination with everolimus for the treatment of advanced renal cell carcinoma on may, 2016. Table 1 shown below represents combination which was approved therapies that have gained FDA approval since 2013 [4].

The approval of the combination drugs over the past few years greatly highlights the potential of using multiple drugs or functionalities towards improving the efficiency for cancer treatment. However, these treatments are detrimental to the normal tissue due to the exposure to the toxic drugs resulting in a lot of short term physical and long term side effects [5,6]. Moreover patients undergoing cancer treatment either chemotherapy or radiation treatment are prone to get affected by infectious disease due to deterioration in immunity [3]. This constituted the idea of encapsulating the toxic drugs in nanoparticles enhancing sustained release of the drugs thereby palliating its side effects during treatment. This led to the postulation of integrating nanotechnology for designing anticancer therapies.

The nanomedicine field designs and develops nanocarriers to transport therapeutic and/or diagnostic agents in human biologics [7]. These nanocarriers are typically less than 200 nm diameter [8]. The field of nanomedicine has rapidly caught interest in designing the cancer therapies mainly because drug delivery systems have likelihood to increase circulation half-life of the drug(s), improve pharmacokinetics, and reduce side effects [9]. Nanoparticles assist in delivery of drugs by taking advantage of the poorly formed vasculature; the enhanced permeation and retention effect (EPR Effect) observed in the cancer environment [10,11]. In addition there has also been a strong advocacy for targeting the vasculature of tumor sites using ligands specific to the type of cancer [12]. The success to this approach was recognized when the FDA approved the first nanomedicine for cancer treatment; which consisted of doxorubicin (DOX) loaded in a liposomal construct (DOXIL) for the treatment of Kaposi sarcoma, ovarian cancer, and multiple myeloma [13].

**Figure 1:**
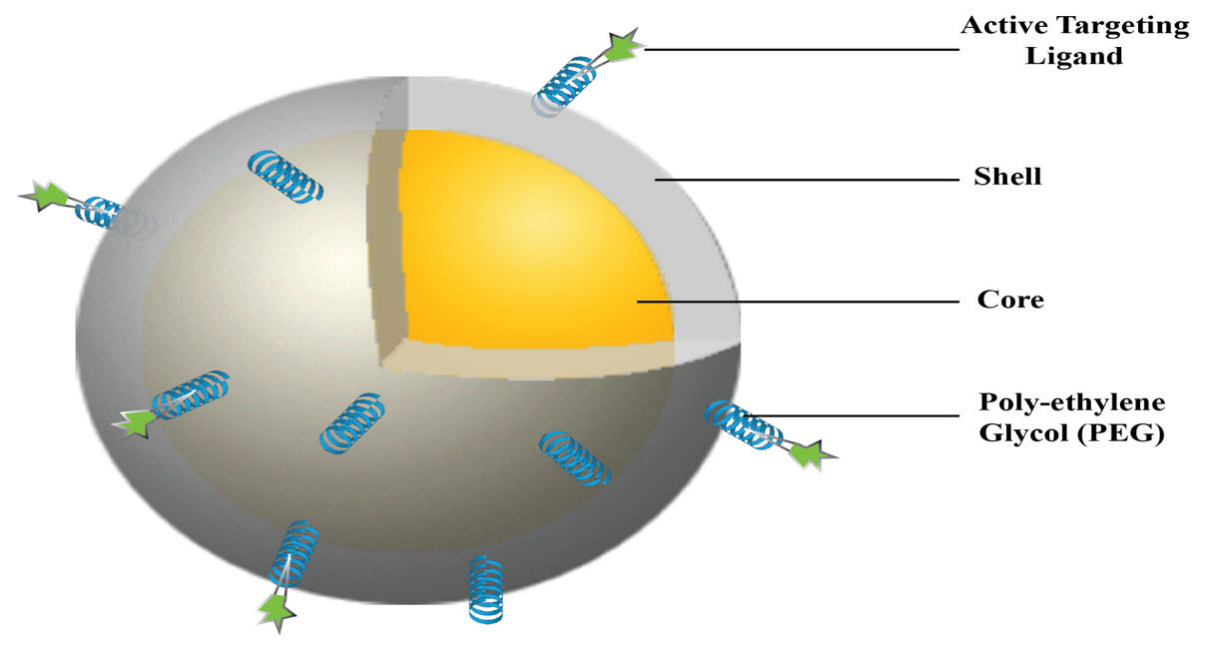
Targeted Nanomedicine- a cartoon showing a representative, targeted stealth nanoparticle as a drug delivery carrier with core-shell structure. A polyethylene glycol (PEG) coating on the nanoparticle surface has shown an improvement in colloidal and storage stability *in vitro* and circulation half life *in vivo* along with providing a spacer for the targeting ligand to bind to its cognate biomarker on the cancer cell.

Nanoparticles are particularly promising drug carriers because of their ability to deliver hydrophobic and/or hydrophilic drug molecules at the tumor site with minimum toxicity to surrounding tissues. Due to their size in nanoscopic scale, nanoparticle carriers are small enough to pass through the leaky vasculature of the tumor, while remaining large enough to escape immediate clearance [6]. The most commonly used nanoparticle carriers for cancer treatment are shown in figure 1. The figure includes both therapeutic and diagnostic carriers with numerous advantages. Liposomes are cell like structures composed of lipid bilayer. The hydrophilic outer layer allows the liposome to move through the human body easily while drugs or markers can be loaded in the core or trans-membrane section of the liposomes [14,15]. The polymeric nanoparticles have high drug loading efficiency, and can be easily chemically conjugated with targeting or therapeutic agents for drug delivery applications. Quantum dots and inorganic nanoparticles such as gold nanoparticles have various applications in photo induced therapy and imaging purposes [16,17]. Dendrimers are branch-like polymeric structures that can house a variety of molecules. The molecules are covalently bonded to the dendrimer, allowing controlled drug release, and thereby making them very effective drug delivery carriers [18].

Nanoparticles have proven to be beneficial towards delivery of the therapeutic drugs and reducing the toxicity; but the treatment to cancer still remains unsolved. For example, Doxil and Myocet (PEGylated and unPEGylated liposomal doxorubicin respectively) show prolonged circulation time and reduction in cardiomyopathy but the overall survival time remained the same as free doxorubicin [19]. In order to improve the efficiency of nanomedicine, a lot of research is carried out in using nanoparticles for combination treatments, using multi functional moiety, for targeting various hallmarks of cancer [20]. While using a multiple drug loading approach with these nanoparticles, the combination of the therapeutic and/or diagnostic drugs selected must compliment each other for the treatment of cancer. Thus it is important to select the correct combination of hallmarks to target and schedule the individual therapies.

**Figure 2:**
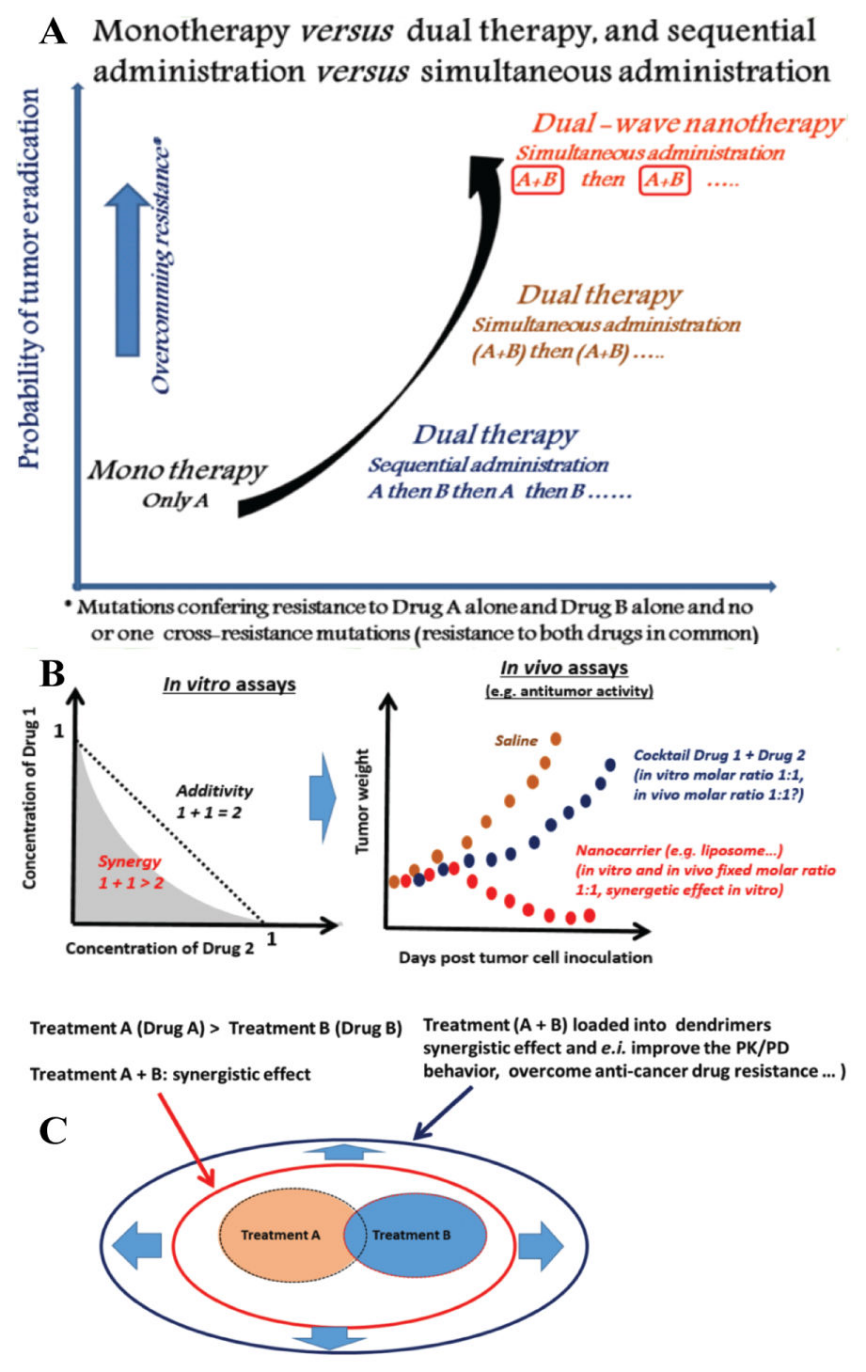
Nanoparticle based combinations therapy. A) Monotherapy versus dual therapy, and sequential administration versus simultaneous administration. B) A schematic representation of cocktail and nanocarrier approaches for combination drug therapy (hypothetical case study shown). The in vitro and then in vivo fixed molar ratio (synergistic effect) can be translated from in vitro assay using nanoparticle approach strategy versus cocktail approach. C) Hypothetical results of a standard clinical trials of two treatments using dendrimers as nanocarrier. Images reproduced with permission from Mignani et. al. [21].

A recent review article by Mignani S. et al. emphasizes the potential of the nanocombination therapy to effectively treat tumors. Relevant information about nanoparticle based combination therapies are highlighted in Figure 2. Several therapeutic molecules, like peptides, small molecule drugs, antibodies are delivered by the nanoparticles for treatment of cancer. Although it is significant to obtain the correct loading efficiency and the release of each of the therapeutic agents from the nanocarrier in order to achieve a synergistic effect. The article also highlights on the potential issues that could surface while using combination treatment using drug delivery agent when transforming from *in vitro* to *in vivo*, and the importance of accurately determining the molar ratio of the drugs for the animal studies within the range of the maximum tolerated dose [21].

The progress in the combination therapies using nanoparticles has been discussed previously in several review articles. Few of them have mainly focused on the type of the nanoparticles used. This review briefly highlights the contributions to combination therapy over the last five years, based on type of cancer treatments. Although review is not exhaustive, efforts were made to be extensive and meticulous in providing detailed information about synergistic therapeutic effect of combination therapy using nanotechnology. These studies using nanoparticles have been divided into possible combinations of cytotoxic therapies (like chemotherapy, photodynamic and photothermal therapies) along with biologics and immunotherapies. The next subsection begins by looking at preclinical studies involving combinations of two or more cytotoxic therapies for cancer treatment.

### Cytotoxic-Cytotoxic Therapy Combinations

**Figure 3:**
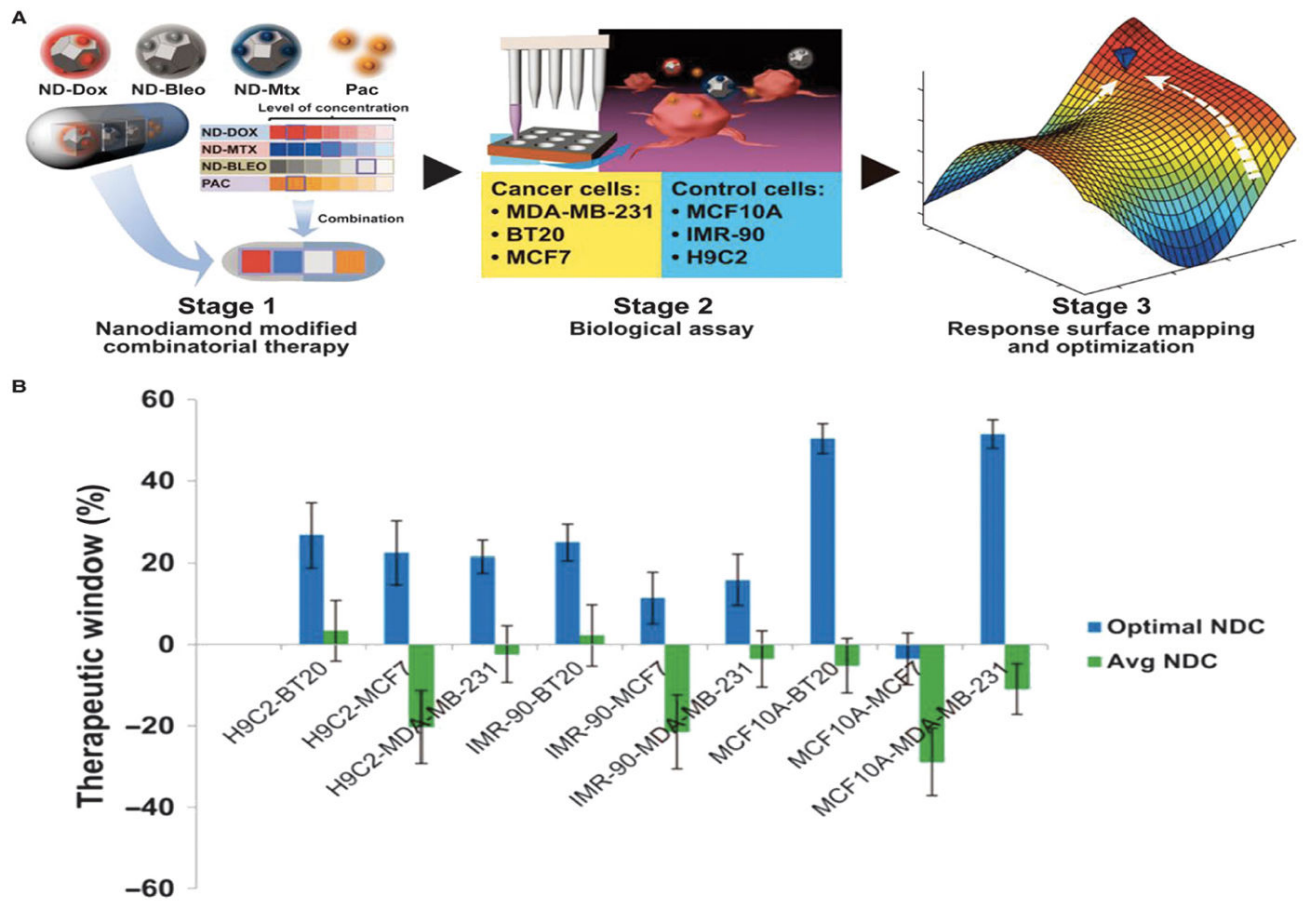
PPM-DD–optimized ND-drug combinations. (A) A schematic model of the PPM experimental framework. Doxorubicin; Bleomycin; Mitoxantrone; Paclitaxel. (B) PPM-derived optimal ND-drug combinations (NDC) outperform a random sampling of NDCs in effective therapeutic windows of treatment of cancer cells compared to control cells. Reprinted with permission from H. Wang et al. [24] Copyright 2014 American Chemical Society.

A wonderful representative example of combinations of multiple cytotoxic therapies is illustrated in Figure 3. In this study a nanodiamond-based drug delivery has been included in the rational and methodical design of optimal therapeutic combinations using an implicitly de-risked drug development platform technology, termed Phenotypic Personalized Medicine–Drug Development (PPM-DD). The application of PPM-DD to rapidly identify globally optimized drug combinations successfully addressed a pervasive challenge confronting all aspects of drug development, both nano and non-nano. In this study, Wang H, et. al. used combination treatment involving a novel nanodiamond as a drug delivery agent for combination sequential therapy for the treatment of cancer. The nanodiamonds were conjugated separately to doxorubicin, mitoxantrone, and bleomycin and tested individually and in combination against the normal cells and cancer cells. The three drugs have the potential to inhibit DNA thereby inducing cell apoptosis when targeted to cancer cells [22,23]. The combination treatment showed greater therapeutic effect against the cancer cells compared. This article also emphasis on the importance of the percentage (%) concentration of each drug required for obtaining synergistic effect, which is the hypothesis of performing combination therapy [24].

### Chemo – Chemotherapy Combinations

Chemotherapy is the most widely clinically practiced treatment for cancer. There are several FDA approved chemotherapeutic drugs that are used specific to the type of cancer and the type of the patient. The different types of chemotherapeutic drugs are classified based on their function and mechanism of action against cancer cells. Most commonly known chemotherapeutic drugs are alkylating agents, antimetabolites, anthracyclines, topoisomerase inhibitors, mitotic inhibitors, and corticosteroids [25]. The choice of selecting two or more chemotherapy drugs mainly depends on factors such as the stage and type of cancer, and drug-drug interaction. During multiple loading two or more drugs in a single nanocarrier, choice of drugs determines whether the effect would be synergistic, additive or antagonistic. Hence it is of extreme importance to ensure the study of the behavior of the drugs prior to deciding them. Here are some examples illustrating how effective multiple loading of chemotherapeutic drugs has shown promising results for cancer treatment.

Cisplatin is a platinum-based chemotherapeutic drug that has potential application against testicular, bladder, ovarian, lung cancer and so on [26]. However, the nephron- and neurotoxicities caused by the use of cisplatin has limited its translation from lab to clinical use, several attempts have been made to reduce the toxicity of cisplatin by encapsulating it in a nanocarrier. In this study, Guo et al., enhanced the encapsulation of cisplatin by coating it with dioleoylphosphatic acid (DOPA), that was further encapsulated in PLGA-PEG-(p- Methoxybenzylamide) along with Rapamycin for treatment against A375-luc human melanoma cells. Rapamycin acts as an mTOR inhibitor thereby promoting antiangiogenesis in tumor environment, and also aids in sensitizing cancer cells to cisplatin by down regulating antiapoptotic proteins such as p53- induced. Cisplatin on the other hand acts as an antiproliferative agent, leading to cell death. During the *in vitro* studies on A375- luc melanoma cells, it was found that the IC_50_ of this nanosystem was 0.3 μM compared to combination of free drugs showing IC_50_ at 0.82 μM. Mice bearing subcutaneous A375-luc tumors showed the least increase tumor volume when treated with combinatory nanoparticle ~250 mm^3^ compared to PBS control which showed the tumor volume increased to ~1250 mm^3^ [3]. In addition to enhanced cytotoxicity, the combinatory nanoparticles also reduced the stromal cells, showed extended penetration into the tumor mass, induced apoptosis of tumor associated fibroblasts and also showed anti-angiogenenesis effect and enhanced normalization to the tumor cells [27].

Many times problems remain in the encapsulation of both the drugs in the same nanocarrier. In this work, Shaik I.M. et al made an effort to enhance the encapsulation of DOX and irinotecan (IRI) in liposomes by using Mn^2+^ gradient coupled with A23187 ionophore. IRI and DOX have the potential to act as topoisomerase I and II inhibitors respectively [28]. Cells require these enzymes to maintain the shape and function of DNA, thus inhibiting it would lead to cell apoptosis. Liposomes were prepared using thin film hydration method followed by extrusion. The dried lipids were hydrated using MnSO_4_/ MnCl_2_ to adjust the pH at 3.5 followed by the extruded samples that were passed a column maintained at pH 7.5. DOX and IRI were loaded post preparation of liposomes by incubating the drugs using the ionophore thereby creating pH gradient. The *in vitro* studies confirmed synergism that at 1:1 DOX:IRI ratio and this ratio was tried for further *in vivo* study was analyzed in the intraperitoneally grown, human IGROV-1 ovarian tumor xenograft model. The study reported 96% increase in life span, showing synergistic results for co-delivery of the chemotherapeutic drugs [29].

The study by Morton S. et al. also designed an experiment consisting of liposomes as nanocarrier encapsulating erlotinib and DOX in the bilayer and core respectively. Erlotinib is an epidermal growth factor receptor inhibitor that helps in activating the capase-8-dependent cell death pathway, which enhances the activity of DOX. The average size of the liposomes obtained was 150 nm and showed a sequential release of erlotinib and DOX. During the *in vitro* studies it was found that % apoptosis of triple negative breast cancer (TNBC) BT-20 and A549 non-small lung cancer (NSCLC) cells when treated with the nanosystem consisting of both the drug encapsulated in liposomes was twice as compared of single delivery of drug in liposomes. In order to target the liposomes to the BT-30 and A549 cells *in vivo*, the nanoparticles were functionalized using PEG-folate. This showed higher cell uptake in both the cells lines along with increased DOX concentration.

The dual drug loading in folate functionalized liposomes showed tumor regression by 32 days whereas the DOX encapsulating liposomes showed continuous tumor growth. In order to test if various other drug combinations can prove useful using the same approach, the core of the liposomes was encapsulated with either DOX or cisplatin, and the lipid bilayer can be encapsulated using either one of these following tyrosine kinase receptors inhibitor; erlotinib, gefitinib, afatinib, or lapatinib. On comparing these combinations with BT-20 and A549 cells, it was observed that the best combination leading to fastest apoptosis for BT-20 cells in vivo was cisplatin-erlotinib, and cisplatin-afatinib encapsulated in folate liposomes where for A549 cells were prone to apoptosis when exposed to the combination involving DOX [30]. These results therefore confirm the importance of selection of the drugs based on the type of cancer.

One of the major issues in treating cancer is multidrug resistance (MDR) offered by the tumor cells that deprives the chemotherapeutic drugs from being efficacious that renders cancer treatment ineffective. The resistance to chemotherapy after initial doses is mainly due to Pglycoprotein (Pgp) and multidrug resistance associated protein that allows tumor regression [31].

Several attempts have been made to overcome this resistance and induce cytotoxicity by using multidrug loading. In one study, PEGylated-phosphatidylethanolamine (PEGPE)/ vitamin E micelles have been used as a nano-carrier to deliver paclitaxel (PTX) and curcumin to SK-OV-3 human ovarian adenocarcinoma cells along with SK-OV-3-PTX resistant cells. The PEGPE/ vitamin E micelles have shown greater bioavailability and higher drug loading capacity. Curcumin helps in down regulating (NF)- κB and AKT-pathway thereby enhancing the efficiency of PTX to induce apoptosis by overcoming the energy dependent efflux of the hydrophobic drugs from the cells. When tested *in vitro* it was found that the drug resistance reversal was best achieved when curcumin concentration was maintained at 10 μM against SK-OV- 3-PTX resistant cells and was confirmed that the optimal ratio of Curcumin: PTX showing synergistic effect was 2.5:1. When the same ratio was used *in vivo* it was observed that combination of both the drugs showed greater tumor growth inhibition for both the cells. The SK-OV-3 paclitaxel resistant tumor volume at the end of 60 days was less than 400 mm^3^ compared to around 700 mm^3^ and 900 mm^3 for delivery of only PTX and curcumin respectively [32].

Another attempt to overcome MDR was by using Verapamil. Verapamil is a first generation ATP binding cassette transporter, proteins over expressed in the tumor microenvironment that actively restricts penetration into tumor mass, blocker that can inhibit the activity of these Pgp and enhance the activity of the drugs. The use of verapamil along with vincristine has shown promising results for the treatment of breast cancer, but the co-treatment showed potential toxicity issues during the clinical studies. Hence to reduce the toxicity, Chen Y, et al. coencapsulated these drugs in PLGA nanoparticles and showed that co-delivery has elaborated the cytotoxicity. The *in vivo* testing showed that the codelivery in same nano-formulation showed 64.04% tumor inhibition efficiency [33]. PLGA Nanoparticles were also used to co-deliver D-atocopheryl polyethylene glycol succinate (Vitamin E TPGS) along with docetaxel to enhance cytotoxicity against multi-drug resistant. Vitamin E TPGS acts as an inhibitor to MDR associated proteins and can be used to enhance the therapeutic efficacy of chemotherapeutic drugs such as docetaxel. Docetaxel is an anti-mitotic chemotherapeutic that interferes with the microtubules thereby preventing cell proliferation and inducing apoptosis. Co-delivery of E TPGS along with docetaxel showed enhanced killing in HeLa cells *in vitro* when 20% of E TPGS was used in E TPGS/PLGA mixture. The IC_50_ value reported was 0.009, showing around 300 times greater killing than free docetaxel. More over when tested *in vivo* the nanomedicine had enhanced accumulation in the tumor site and showed synergistic killing confirming the use of 20% ETPGS codelivered with docetaxel using PLGA nanoparticles for cancer treatment [34].

Another example emphasizing on MDR is combination treatment using DOX and disulfiram, P-gp inhibitor, encapsulating pH sensitive polymeric micelles nanosystem. The pH sensitive nanosystem comprise of disulfram loaded poly (styrene-co-maleic anhydride)- adipic dihydrazide conjugated DOX. This combined chemotherapy showed around 82% reductions in the tumor weight, confirming to be a promising intracellular delivery system for cancer treatment [35]. In this study a novel nanoscale coordination polymer-1 (NCP-1) was used to codeliver oxaliplatin and gemcitabine monophosphate (GMP) for treatment of pancreatic ductal adenocarcinoma. Gemcitabine and oxaplatin inhibits DNA replication thereby obstructing the process of cell proliferation [36,37]. The codelivery of these drugs using NCP-1 showed enhanced antitumor activity with approximately 75% apoptosis for pancreatic cell lines *in vitro*, and confirmed synergistic effect leading to tumor weight inhibition at lower drug concentration showing ~77.8% of apoptosis when tested *in vivo* [38].

Wang. et al. utilized a modified double emulsion (w-o-w) technique to synthesize nanoparticles capable of simultaneous encapsulation of drugs with different water solubility. The amphiphilic copolymer mPEG-PLGA was used to form core shell droplet particles with an inner aqueous phase for delivery of hydrophilic drugs, such as DOX in this study. The inner phase is surrounded by a hydrophobic region for delivery of water insoluble drugs, represented by PTX in this study. The codelivery nanoparticles were found to induce cytotoxicity more effectively than free drug at the same concentrations in A549, HepG2, and B16 cells [39].

Wang. et al. also utilized poly (ethylene glycol)- block-poly (2-methyl-2-benzoxycarbonylpropylene carbonate) (PEG-PBC) copolymer to prepare micelles for combination delivery. In this study, both DOX and lapatinib were encapsulated in the core of the particle for combination delivery to MCF-7 and MCF-7/ADR cells. Intracellular accumulation was significantly enhanced by the micelle formulation, as compared with free drug, in the MCF-7/ ADR cells overexpressing Pgp. The micelle vehicle also showed increased effectiveness during *in vivo* studies against tumors established with MCF-7/ADR cells in mice [40].

Current combination treatment is limited by the distinct pharmacokinetics and biodistribution profiles of different drug molecules. Ideally, synthesis of combination vehicles would be able to deliver multiple drugs in a precise, tunable ratio. Zhang et al. synthesized DOX and camptothecin polymer conjugates for encapsulation in lipid polymer hybrid particles. The group achieved consistently high encapsulation (>90%) for single drug and dual drug vehicles, hypothesizing that the long PLA polymer chain gives each drug a uniform hydrophobic property. The dual drug vehicle consistently showed higher potency in *in vitro* studies with MDA-MB-435 cells [41].

### Photodynamic Therapy-Photothermal Therapy Combinations

Phototherapy is a form of medical treatment that uses light of specific wavelength to treatment various infectious diseases. There are mainly two types of phototherapy: photodynamic therapy (PDT) and photothermal therapy (PTT) and are extensively studied for cancer treatment [42]. Photothermal therapy converts photon energy into heat energy to destroy cancer cells. PTT is carried out in presence of a photothermal (PT) agent that helps in selective heating at the tumor site leading to denaturation of proteins and disruptions of cell membrane causing cell death [23,27,43,]. PDT is a treatment, which uses a photosensitizer (PS) that is administered to the tumor site and selectively irradiated with laser light of specific wavelength based on the PS. This results in generation of reactive oxygen species that can induce cytotoxicity to tumor [44]. This section mainly focuses on the combination of loading a chemotherapeutic drug along with either PT agent or PS to induce phototherapy.

Nanocarriers have enhanced the capacity of inducing phototherapy towards treatment of cancer. In their research, Chen H., et. al. showed how nanoparticles carrying PS can absorb low doses of deeply penetrating X-rays exhibiting the potential to carry PDT deeper into tissues [45,46]. In this section we have some examples elaborating on use of either a nanocarrier that can play a dual role of delivering drug and acting as PS/PT agent, or delivery of a drug that can play a dual role of being PS and PT agent both.

Nano graphene oxide (GO) sheet has attracted attention both as drug delivery and PT agent. In a recent study, Sahu A., et al. used pluronic coated nano GO sheet mediated delivery of methylene blue (MB) at the tumor environment. The approach behind the research was to induce combined PTT and PDT using graphene oxide as PT agent and methylene blue as PS. The nano GO sheets were around 45 nm in size and showed ~23% MB loading content. The cell uptake studies showed higher fluorescence of Cy5.5 labeled pluronics 127 coated nano GO sheets by HeLa cells (cancer) more than the NIH/3T3 cells (normal). The *in vitro* studies showed around 20% cell viability of nano GO-MB when treated with combined therapy compared to individual phototherapy (PDT for nano GO-MB and PTT for nano GO). The *in vivo* studies confirmed higher concentration of nano GO-MB accumulated at the tumor site, and the combined PDT-PTT treatment showed complete regression of tumor post 15 days at lower nano GO dose, which was not seen in the individual therapy [47].

A multifunctional magnetic and fluorescent graphene (MFG) nanosystem was designed to treat and diagnose cancer. These MFG nanoparticles were non-covalently functionalized with napthalocyanine bis (trihexylsilyloxide) (SiNc_4_) that acts as a PS and also increases biocompatibility of MFG. This MFG- SiNc_4_ can be used as a theranostic agent, and induce PDT and PTT at a single wavelength. The fluorescence induced from MFG and SiNc_4_ shows that the nanoparticles were internalized in HeLa cells after 24 hours incubation time. Being supramagnetic in nature, MFG can act as MRI contrast agent hence can be used for diagnosis while the treatment. The advantage of this system is that by immobilizing SiNc_4_ onto grapheme, only a single laser source at 775 nm could be used to induce PDT and PTT at the same time resulting in destruction of cell proteins and membrane due to PDT and tumor ablation due to PTT thereby minimizing the chances of tumor recurrence. The reported highest cell death obtained was 97.9% at 0.1 μM of SiNc_4_ and 10 μg/mL of MFG- SiNc_4_ [48].

Another study highlights the use of phthalocyanine encapsulating hollow silica nanoparticles (Pc@HSN), a multi-functional system that can prompt both PDT and PTT. Pc@HSN were illuminated with 730 nm laser light for both PDT and PTT. *In vitro* studies showed maximum killing at 400 μg/mL Pc@HSN concentration when KB cancer cells were irradiated with 730 nm laser at 1.5 W/cm^2^ power densities for 8 min. When the nanoparticles were injected in malignant tumor cell line S180, the tumor tissue was completely destroyed after 5 days. This clearly proves the efficacy of dual phototherapy for treatment of cancer [49]. Gao L. et. al used lipid- loaded hypocrellin B, photosensitizer, to coat Au nanocage to induce combined PTT and PDT *in vitro* on HeLa cells. The treatment was performed by irradiating 790 two photon laser at 85.5 pJ per pulse for 300s and showed 17.4 % cell viability confirming synergistic anticancer therapy [50].

### Chemotherapy-Phototherapy Combinations

The treatment of metastatic breast cancer was proposed by using dastinib, an anti-migration drug was loaded in albumin that formed shell to PLGA nano-core encapsulating mtetra (hydroxyphenyl) chlorin (mTHPC), a photosensitizer. PLGA-albumin core-shell encapsulating the drugs showed hydrodynamic size of ~100 nm, with 44% of mTHPC loaded in PLGA nano-core and 94% dastinib loaded in albumin shell. On testing the cell migration capacity of metastatic breast cancer cell line MDA-MB-231 and MCF-7 using dastinib loaded albumin-PLGA core-shell nanoparticle, it was observed that at 5 μM dastinib concentration both the cell lines showed reduced migration potential. During the *in vitro* studies, the PDT showed enhanced killing to ~98% when the concentration of mTHPC in the PLGA core was 2.5 μM and dastinib concentration in the shell was maintained at 5 μM showing synergistic effect in inducing cytotoxicity against metastatic breast cancer cells [51]. Another example for nano core-shell system comprised of biodegradable poly (ε-caprolactum) -poly(ethylene oxide) (PCLPEO) block copolymer co-delivering chemotherapeutic drug docetaxel and photosensitizer Zn (II) phthalocyanine. The *in vitro* study showed around 90% death of HeLa cells while and *in vivo* studies confirmed greater % survival rate of mice treated with this combination therapy [52].

Fullerene is another nanoscale carbon material (C60) that can be used as a PS and a drug delivery carrier. In a recent study, DOX was delivered to tumor site when loaded with poly(ethyleneimine) (PEI) conjugated fullerene. This system induced controlled pH responsive drug release via cleavage of hydrazone bonds between C60-PEI and DOX. At acidic pH 5.5, drug release at 37°C was around 86.2% after 60 hours compared to 14.1% at pH 7.4. The PDT experiment showed that at 4 μg/mL DOX concentration, C60-PEI DOX NPs showed around 94.2% killing efficiency of B16-F10 cells when irradiated with 532 nm laser, which was significantly high compared to DOX free C60-PEI with 532 nm laser. The *in vivo* studies also confirmed tumor reduction when mice bearing B16-F10 tumor were treated with C60-PEI DOX NPs and irradiated with laser at 532 nm thereby proving the therapeutic efficiency of C60-PEI DOX for cancer therapy. Nano core shell polymeric nanoparticles carrying cisplatin and a photosensitizer pyrolipid was used to test combination chemotherapy and photodynamic therapy to treat head and neck cancer. Combination chemotherapy and photodynamic therapy confirmed enhanced cytotoxicity compared to the individual DOX treatment showing 74.8% cell death [53].

In this study, PEGylated polyamidoamine (PAMAM) G4 dendrimer was conjugated to Au nanorods, which was further conjugated to DOX to the terminal layer of the dendrimer to induce chemophotothermal therapy. During the *in vitro* studies with HeLa cells, the IC_50_ of DOX conjugated PEG-PAMAM-AuNR was observed at 13.7 μg/mL compared free drug, which killed more than 98.5% of cancer cells. When irradiated with 808 nm laser source at 6 W/cm^2^ for 10 mins, the IC_50_ dropped to 2.2 μg/mL, similar to that of free DOX. For examining the antitumor activity *in vivo*, BALC/c mice were inoculated with mouse colon carcinoma 26 cells on both the flanks. It was found that PEG-DOX-PAMAM-AuNR treated mice showed elevated temperature to when irradiated with light at the tumor site but showed no significant change in temperature in the neighboring tissue. Also initially PEG-DOX-PAMAM- AuNR showed similar reduction in tumor growth as PEG-PAMAM-AuNR but showed enhanced growth delay post day 16 showing efficiency for a potential chemo-photothermal therapy [54]. Zheng M et al. used doxorubicin/ indocyanine green loaded folate lipid PLGA nanoparticles combination chemo phototherapy. This combination treatment showed increase in cell death *in vitro* and complete tumor eradication with no tumor relapses *in vivo*. This treatment induced synergistic cell death for both DOX sensitive and DOX resistant cancer cells, proving to be a promising combination therapy for treatment of cancer [55].

Yang HW. et al. used combination of chemo-photothermal therapy to demonstrate tumor suppression by using targeted nano graphene oxide. Nano GO carrying epirubicin was conjugated with epidermal growth factor receptor (EGFR) antibody C225 to target against gliomas. The *in vitro* studies confirmed that C225 when conjugated with PEG-nano GO does not only target the glioma U87 cells but also inhibits EGFR expression. The cytotoxic studies showed that the IC_50_ of the nanosystem comprising of epirubicin encapsulated nano GO conjugated with C225 was only 2.7 μg/ mL when exposed to 808 nm laser at power intensity of 2W/ cm^2^ for 120s compared to 9.7 μg/mL without exposure to laser. When the NPs were test on mice, it was found that tumor ablation was observed by 10 days when laser was irradiated 3 days post injection. There was no relapse in tumor observed after 30 days proving that this system has the potential to not only cause tumor ablation but also inhibit tumor recurrence [56].

In more recent study, Xue P, et al. highlights the development and potential use of new class of PT agents; Prussian blue nanoparticles, to induce PTT along with delivery of doxorubicin to enhance cell killing efficacy of the nanosystem. Prussian blue nanoparticles were coated with DOX conjugated gelatin (PB@Gel-DOX NPs) to stabilize the NPs and to induce enzymatic responsive drug release thereby lowering the toxicity in the normal tissue. During the PTT *in vitro* studies, PB@Gel-DOX NPs were treated with HuH7 cells. It was also confirmed that without laser irradiation, the release of DOX from PB@Gel-DOX NPs showed up to 25% cell killing in HuH7 cells at 80 μg/mL NPs concentration. When irradiated with laser at same concentration, the cell viability reduced to ~ 10% showing synergism in the chemo-photothermal treatment [57]. A novel multifunctional iron oxide-polypyrrole nano core-shell system functionalized with doxorubicin was used as drug delivery carrier for combined photothermalchemotherapy. PEGylated iron oxide-polypyrrole nano core-shell system exhibited properties of potential PT agent at 808 nm, and showed temperature increased to 44–45 °C. The combined PTT and chemotherapy using doxorubicin showed synergistic effect in treating cancer, and completely eliminated tumor post *in vivo* treatment [58]. Ma. et al. prepared nanoellipsoids using Au nanorods covered by mesoporous silica shell and further loaded with doxorubicin for a combined photothermal chemotherapy. The optimal concentration of nanoellipsoids and doxorubicin was identified as 60 μg/mL and 25 μg/mL respectively, and synergistic cell killing was showed for *in vitro* and *in vivo* studies using MCF 7 cells [59].

**Figure 4:**
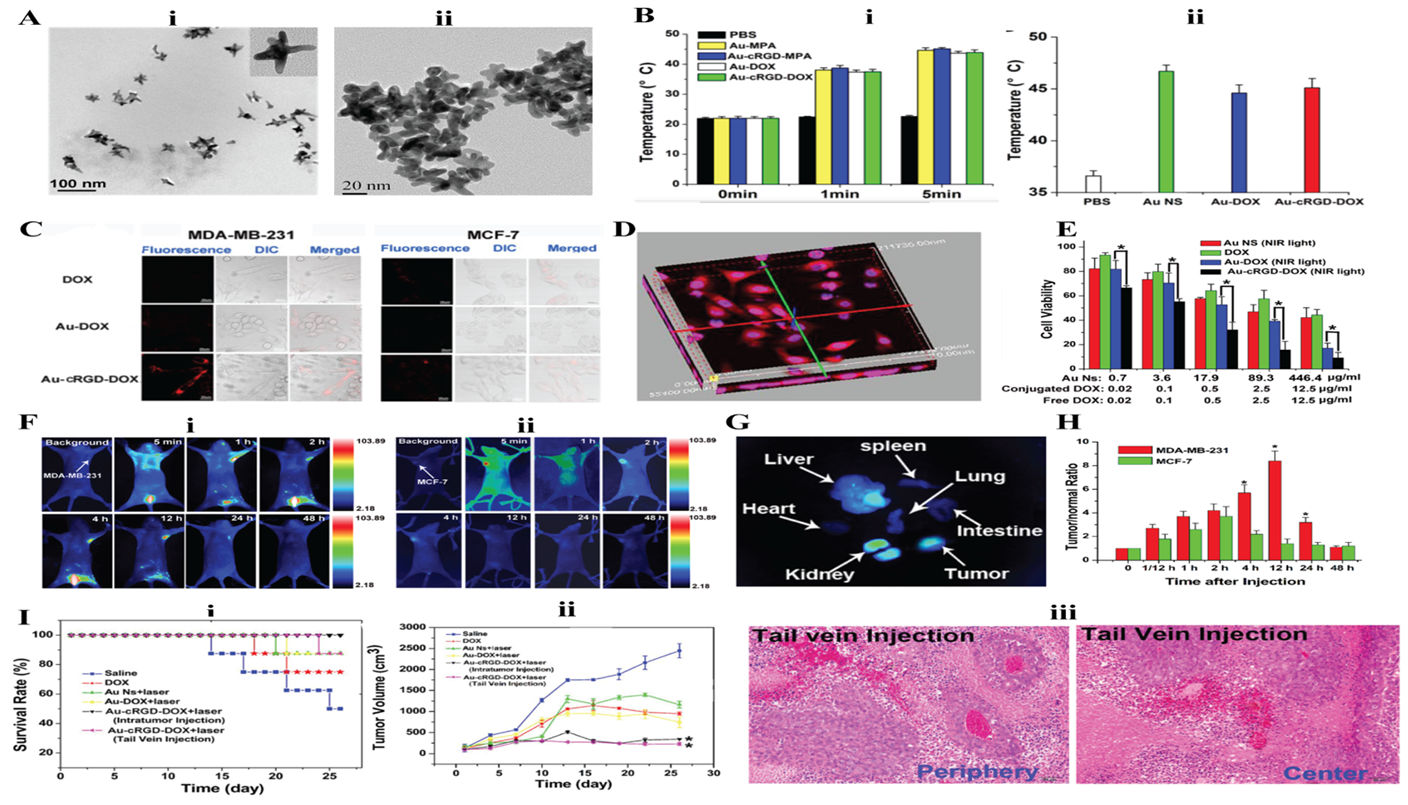
Gold Nanostars based theranostics for combined chemo- and photothermal therapy **A)** TEM Images showing the morphology of Gold Nanostar, before cRGD conjugation (i) and after cRGD conjugation (ii). **B)** Temperature of PBS, Au-MPA, Au-cRGD-MPA, Au-DOX, and Au-cRGDDOX upon light irradiation in vitro (i) and in vivo (ii). **C)** Intracellular uptake of DOX, Au-DOX, Au-cRGD-DOXMDA-MB-231 and MCF 12A cells after incubation for 1h. **D)** 3D fluorescence image of Au-cRGD-DOX internalized by MDA-MB-231 and transported to nuclei. **E)** Qualitative killing analysis of tumor activity in vitro of Au-NS, DOX, Au-DOX and Au-cRGD-DOX on MDA-MB-231. **F)** In vivo images of Au-cRGD-MPA in MDAMB- 231(positive αvβ3 receptor expression) (i) and MCF-7 (negative αvβ3 receptor expression) (ii). **G)** The ex vivo fluorescent images of individual organs. **H)** Tumor-to-normal tissue (T/N) ratios of Au-cRGD-DOX in MDA-MB-231 and MCF-7 tumor cell line. **I)** Comparison of the therapeutic efficacy of Au-NS +light, free DOX, Au-cRGD-DOX+light (intratumoral injection and Au-cRGD-DOX light (Tail vein injection) in S180 tumorbearing mice. Survival rates of mice (i), tumor growth of mice (ii), H&E stained tissue section for tail vein injection (iii). Images reproduced with permission from Chen, et al. [60].

In a preclinical study study Chen. H. et al. focused on synthesizing gold nanostars (Au NS) as drug delivery carriers for cancer treatment and imaging. Relevant results of this study have been reproduced in Figure 4. Au NS were functionalized with doxorubicin and RGD peptide to induce combined photothermal and chemotherapy. Au is a known photothermal agent, while RGD peptide binds to the RGD receptor and plays a critical role in regulating tumor angiogenesis, and DOX brings about apoptosis. The photothermal treatment showed increase in temperature to 43.9 °C both *in vitro* and *in vivo* studies when irradiated with 1W/cm^2^ laser light at 765 nm. The *in vivo* cell uptake studies were performed to confirm the specificity of the RGD peptide towards over expressing integrin αvβ3 cell line like MDA-MB-231. The multifunctional AuNS also showed enhanced antitumor activity, and lowest cell viability upon irradiating light *in vitro. In vivo* studies showed that mice treated with Au NS via intravenous injection showed 100% survival. Au NS was also tested via tail vein injection but did not prove to be as efficient as intravenous [60].

## Cytotoxic–Biologics Combination Therapies

### Chemotherapy-Immunotherapy Combinations

It has been studied that tumor cells have mechanism to evade immune system, whereas chemotherapy is well studied for tumor treatment. A combined chemo-immunotherapy approach could be an efficient strategy to increase the therapeutic efficiency against tumor.

The immune system has native capacity for suppressing tumor activity and proliferation within the body. Tumor cells recognized by the host immune system elicit a response from the innate and adaptive immune systems. The immune system will attempt to clear the tumor cells from the body or establish an equilibrium where the tumor is monitored and growth is inhibited. It has been established that some chemotherapeutic regimens enhance the innate immune response by targeting individual steps within the response. A typical response may consist of antigen presenting cells (APCs) relaying tumor antigens to tumor-specific T cells, which then differentiate into effector T cells. For an effective response, it is necessary that the T cells avoid negative regulatory signals from the host and overcome any immunosuppressive networks within the tumor microenvironment [61]. Treatment options can target any step within the response chain. For instance, mild or low dose chemotherapeutic agents may decrease the effectiveness of regulatory T cells and myeloid-derived suppressor cells [62]. Current immunotherapy struggles to account for the complexity of the immune system – tumor relationship. The myriad immune cells involved in a tumor response necessitate stimulating therapy targeting numerous processes. Monotherapy is unlikely to provide the necessary stimulation across cell types and signaling pathways. Combination drug delivery may provide the more diverse therapy essential for allowing a potent anti-tumor immune response.

In one study, PLGA NPs have been used to co-deliver PTX and phthalate ester of lipopolysaccharide (P-LPS) [63]. Lipopolysaccharide is a toll-like receptor 4 agonist that activates dendritic cells and tumor associated macrophages to enhance the immune response. The release kinetics showed sequential release of P-LPS followed by PTX, showing 80% and 35% respectively after 48 hours. The NPs showed increase in immune response, which was confirmed by increase in T helper cell 1 (Th1) cytokines. The *in vivo* study on C57BL/6 mice inoculated with B16-F10 melanoma, showed significant reduction in tumor volume and mass when the concentration was maintained at 10 mg equivalent PTX per kg. The immune response was verified by analyzing the infiltration of the immune cells in the tumor tissue. An increased expression of APC and T cell in the tumor microenvironment was observed confirming the potential use of combined chemo-immunotherapy for treatment of cancer.

Another study emphasized the use of combined chemo-immunotherapy for treatment of B16-F10 melanoma by using poly (γ-glutamic acid) to co-deliver PTX and imiquimod, a toll like receptor 7 agonist used to stimulate immune response for cancer treatment [64]. This study showed significant tumor killing efficacy *in vivo* and increased activation and maturation of dendritic cells in the tumor microenvironment.

Cell receptors overexpressed by tumor cells are also attractive targets for nanoparticle delivery. Molavi. et al. delivered Doxil by targeting CD30, a member of the tumor necrosis factor receptor superfamily overexpressed in anaplastic large cell lymphoma (ALCL) [65]. The group conjugated anti-CD30 monoclonal antibodies (mAbs) to PEG-coated Doxil. The average diameter of the particles was found to be about 87.4 nm. The final concentration of DOX in CD30-targeted Doxil was 0.26 mg/mL, compared with a concentration DOX in Doxil of 2.0 mg/mL. The CD30-targeted Doxil exhibited significantly higher binding than non-targeted Doxil. The *in vitro* cycotoxicity of the two groups was also compared. The authors found that the IC_50_ of non-targeted Doxil is approximately three times higher than that of CD30-targeted Doxil and this was statistically significant.

*In vivo* study in a SCID mouse xenograft model allowed therapeutic efficacy evaluation. Mice were treated with either CD30-targeted Doxil or non-targeted Doxil once tumors reached 0.1 cm3. After four doses, average tumor size in mice treated with CD30-targeted Doxil was significantly lower than mice treated with non-targeted Doxil. In addition, treatment with non-targeted Doxil resulted in 14.3 ± 1.9% decrease in mouse body weight, while mice treated with CD30-targeted Doxil were found to have no change in body weight compared with the control group.

Antibody-antigen coating is also used to stimulate the host immune system. Li et al. studied bufadienolides loaded liposomes (BFL) coated with polyethylene glycol (PEG) and conjugated with CD40 targeting antibodies [66]. BFL exhibits antitumor activity through induction of apoptosis, cell cycle disruption, and immune response regulation. CD40 is a member of the tumor necrosis factor (TNF) receptor superfamily present on numerous immune system and APC. The receptor enhances the capability of APCs to present antigens, resulting in enhanced antitumor cytotoxic T-cell responses. The prepared liposomes were characterized and their effectiveness evaluated both *in vitro* and *in vivo*. Anti-CD40- BFL liposomes had an average diameter of 205.4±68.4 nm, while the average diameter of non-targeted BFL only liposomes was 171.4±69.2 nm. Surface concentration of anti-CD40 mAbs on the liposomes was estimated to be 5 mg/mL by ELISA analysis.

*In vitro* cytotoxicity was evaluated using B16 cells. BFL and anti- CD40-BFL liposomes exhibited greater cytotoxicvity on the B16 cells than free bufalin. The two liposomes showed similar inhibitory effects, which the authors attribute to B16 cells lacking CD40 surface expression. Anti-CD40-BFL liposomes also exhibited superior growth inhibition *in vivo*. C57/BL6 mice bearing B16 cell tumors were treated with saline solution, Bufalin solution, anti- CD40 solution, BFL liposomes, and anti-CD40-BFL liposomes. After five treatments, all treatment groups exhibited tumor growth inhibition, though the anti-CD40-BFL treatment group showed the greatest response. Average tumor volume in this group was 176 ± 43.86 mm^3^ versus 254.53 ± 44.99 mm^3^ in the BFL liposome treatment group (P=0.0482).

Viral vectors also possess the potential to stimulate the immune system. Cao et al. delivered liposomes loaded with PTX and adenoviral vector expressing interleukin-12 (Ad5- mIL-12) [67]. IL-12 is produced by APC and induces numerous antitumor effects. Liposomes containing adenovirus vectors (Ads) and PTX were synthesized by calcium-induced phase change. Particles containing both adenoviral vector and PTX were measured to be 242.2 ± 5.07 nm diameters and −27.5 ± 1.39 mV zeta potential.

The antitumor efficacy of combinations of PTX and Ad5-mIL-12 loaded liposomes was evaluated *in vivo* using B16 melanoma tumor-bearing mice. Tumor growth rate in mice treated with either PTX or Ad5-mIL-12 loaded liposomes was significantly lower than the control group treated with PBS. The combination treatment of Ad5-mIL-12/PTX loaded liposomes showed the highest therapeutic effect.

Another example highlights the effect of combination of chemo-immunotherapy for treatment of melanoma. In this study, polylactic acid was used as drug delivery agent to carry dacarbazine (DTIC) [68]. DITC is an alkylating agent that binds to the DNA of cancer cells inducing programmed cell death. DTIC loaded nanoparticles were conjugated to anti-tumor necrosis factor related apoptosis inducing ligand (TRAIL) –receptor 2 DR5 antibody to specifically target the malignant melanoma cells. When these nanoparticles were tested on DR5 positive A375 cells, the cell uptake studies showed increased internalization via ligand-mediated mechanism compared to insignificant binding to DR5 negative NIH cells showing high specificity of the targeting agent. This verified that the NPs were more cytotoxic to A375 cells compared to NIH cells. During the evaluation of cell apoptosis, the results indicated that DTIC encapsulated DR5-PLA NPs has high apoptotic efficiency confirming that this nanosystem can be used as an efficient targeted delivery system against melanoma cells [69].

### Phototherapy-Immunotherapy Combinations

An effort was made towards treating ovarian cancer by inducing a PDT along with immunotherapy. The co-delivery of Cetuximab (C225 antibody), against epidermal growth factor receptor and a photosensitizer, benzoporphyrin derivative monoacid A (BPD) to perform PDT was done by physically adsorbing them on preformed plain liposomes. The release kinetics showed that ~15% of BPD and ~41% of C225 antibody was released after 24 hours when incubated in media. This was explained to be advantageous as C225 can induce biological action, thereby supplementing the PDT treatment with BPD-liposomes. The *in vitro* results also showed significant uptake of BPD by Ovcar-5 when delivered using C225 adsorbed liposomes. The PDT was performed at 250 nM BPD concentration, and 3 μg/mL of C255. The % cell viability of this nanosystem-based PDT when irradiation was done at 10J/cm^2^ was only ~7% compared to free BPD which was around 56% [70].

### Photo-Immuno-Chemotherapy Combinations

An attempt has been made towards designing a combination therapy for treatment of cancer constituting an immune-stimulatory toll like receptor agonist, unmethylated cytosine-guanosine (CpG) oligodeoxynucleotides (ODNs) conjugated to gold nanorod, whereas DOX, a well known chemotherapeutic drug, is intercalated into the CpG ODNs. This nanosystem offers localization of gold rods into the tumor site that can be used to induce PTT; the CPG ODNs induce immune response thereby enhancing the cytotoxic effects of DOX. The gold nanorods with sufficient % conjugation of the ODNs and DOX had hydrodynamic size of ~91.5 nm. During the *in vitro* photothermal experiments the % cell viability reduced to 12% when cultured with H22 cell line, whereas significant immune response was observed when H22 cell line was co-cultured with RAW264.7 cells (macrophage) when treated with CpG ODNs conjugated gold nanorod and DOX (ACD). The immune response was quantified based on cytokines levels. The *in vivo* results showed localized heating due to presence of the gold nanorods and showed significant decrease in the tumor volume after 12 days when mice treated with ACD showing the synergistic effect of using combination of chemotherapy, photothermal therapy and immunotherapy [71].

## Biologics-Biologics Therapy

### Immuno-Immunotherapy combinations

The body’s immune system plays a dual role in suppressing tumor progression by recognizing cancer cells as foreign and promoting tumor by tolerating its growth [72]. This is well explained by the concept of cancer immunoediting: a process consisting of immunosurveillance and tumor progression that proceeds in three discrete phases: elimination, equilibrium, and escape [73]. During the elimination phase, the innate and adaptive immune cells recognize the transformation of cells into developing tumor cells and destroy them. A few of the cancer cells pull through the elimination phase to enter the equilibrium phase where the adaptive immune cells prevents tumor growth and maintains cancer cells in the state of dormancy. In the escape phase, the tumor cells evade immune surveillance and can even grow in immunocompetent environment [74]. Tumor cells derails immune response by secretion of immune suppressive signals [9,75]. Although tumor cells get less immunogenic, there are many known tumor-associated antigens that can play as targets to design therapies that can enhance immune response for cancer treatment [76]. Cancer immunotherapy has received rapid recognition and has shown promising results towards cancer eradication. In spite of showing promising results, there are several shortcomings of cancer immunotherapy. For instance, Provenge, an autologous dendritic-cell vaccine helps in improving the survival rate by 4.1 months only and costs $93,000 for the treatment being one of the most expensive cancer therapy [77,78]. Moreover, immunotherapy is not yet preferred as the first line of treatment. While chemotherapy, the first line of treatment; often weakens the immune system the eleventh-hour treatment to intensify immune response does not prove beneficial. Moreover the traditional immunotherapeutic formulations might have early clearance of the pathogen/antigen resulting in premature degradation [79]. This can be prevailed by using nanoparticles to deliver antigens that can lead to elevated effector and memory response at reduced drug dosage [80]. The primary role of using nanocarriers would be to enhance specific immune activity against cancer, i.e. by presenting antigen to the APC such as dendritic cells that could initiate cyotoxic T lymphocyte mediated response. This section mainly focuses on administrating multifunctional nanoparticles comprising of these antigens can trigger immune response leading to tumor destruction.

This work highlights the use a non-toxic hybrid liposomal polymeric system like core shell nanoformulation (nLG) to deliver transforming growth factor-β (TGF-β) inhibitor SB505124 and interleukin-2 (IL-2). TGF-β is a cytokine that inhibits the activation of immune cells such as natural killer (NK) cells of cytotoxic T (CD 8+) cells, whereas IL-2 is an immunostimulating cytokine. The SB and IL-2 nLG was used to enhance immune response against B16/B6 mouse model of melanoma. SB, a hydrophobic small molecule was conjugation with β-cyclodextran (β-CD) to achieve sustained release of SB and IL-2, which is a water-soluble 17kD large protein molecule. The co-delivery of SB and IL-2 using nLG induced increase in innate (NK cells) and adaptive (CD8+: Tregulatory cells) resulting in inhibiting tumor growth and increase in %survival rate of mice [81].

### Chemotherapy and Gene Therapy Combinations

Nanocarriers are being used to co-deliver chemotherapeutic agents and gene therapy in the form of interfering RNA (RNAi). Most often gene therapy is proposed as a tool for overcoming multidrug resistance (MDR). In 2013 results from the first in human clinical trials of RNAi encapsulated in lipid nanoparticles demonstrated the treatment was generally well tolerated by patients with advanced cancer [6,82].

Cancer cells cope with chemotherapeutic treatment in two ways. First, cancer cells increase gene expression for components of drug efflux pumps, such as Pgp. These pumps bridge the nuclear membrane or cellular membrane and more cytotoxic agents away from the cell environment. In addition, cells cope with chemotherapy by increasing gene expression for antiapoptotic pathways. Gene therapy is able to enhance chemotherapy by interfering with those genes involved in developing chemotherapeutic resistance. Forms of RNAi include small interfering RNA (siRNA), micro RNA (miRNA), and short hairpin RNA (shRNA). MiRNA occurs naturally in most eukaryotic cells, where it is transcribed in the nucleus. This primary transcript (pri-miRNA) can be processed by Drosha to form pre-mRNA. The pre-mRNA is exported to the cytoplasm where it may undergo celavage by DICER gene or endoribonuclease Dicer gene and then enter the general pathway for RNAi silencing, described below. siRNAs are 19–23 nucleotide long double-stranded RNA with 2-nt-3’ overhang. siRNAs are chemically synthesized analogues of miRNA. siRNA enters the gene expression silencing pathway where DICER cleaves the double-stranded RNA. shRNA has been implemented to bridge the deficiencies of siRNA, namely the short half-life of siRNA. shRNA is transcribed within the nucleus from an external expression vector. Transcribed shRNA may then undergo similar processing as miRNA.

The mechanism of gene expression silencing is similar for all types of RNAi, though the exact pathway differs based on the form of RNAi. Within the cell double-stranded RNA can be recognized and processed by the RNA-induced silencing complex (RISC). RNAi is unwound by the Aronaute protein (AGO2) portion of RISC. One strand guides the RISC complex to complementary or near-complementary target mRNA. Gene expression is downregulated by degrading the mRNA or blocking mRNA translation.

RNAi has been used to downregulate major vault protein due to the observation that major vault protein (MVP) is over-expressed in several Pgp negative chemoresistant cancer cell lines [83,84]. MVP is associated with the removal of drug from the cell nucleus, a target of many chemotherapy drugs. DOX – MVP-siRNA dendrimers were delivered to MCF-7/ADR cells [79]. Carriers with siRNA were found to have much more cytotoxic activity than DOX only dendrimers. Imaging was used to verify enhanced accumulation of DOX within the nucleus when delivering DOX-MVP- siRNA, as compared with DOX solution and DOX only dendrimers. The authors also pretreated cells with MVP-siRNA, finding that cells were sensitized to later DOX delivery.

Another study targeting Pgp delivered DOX and Pgp siRNA via mesoporous silica nanoparticles (MSNP) to cells from the MDR breast cancer cell line MCF-7/MDR [80]. A synergistic effect was observed when DOX and Pgp siRNA delivered via MSNP. Greater tumor growth inhibition was observed using the DOX-siRNA loaded particles, as compared to free DOX or the particle with DOX or siRNA alone (inhibition of ~80% compared to 62% with DOX alone carrier). Pgp siRNA alone was observed to have no significant tumor inhibition in the xenograft mice model used. Imaging was used to determine the location of DOX, whether it was ending up at tumor or not. Immunoblotting was used to measure Pgp expression after treatment, verifying effective knockdown by Pgp siRNA. Expression was less than half that of the control group or the scrambled siRNA control group.

The tumor microenvironment is often characterized by an imbalance of proangiogenic and antiangiogenic factors which may be down-regulated through gene therapy. Vascular endothelial growth factor (VEGF) is one such target that has been successfully silenced through anti-VEGF siRNA (siVEGF) [85]. Polyethylenimine-3- maleimidopropanionic acid hydrazide (PH) conjugated with DOX (PHD) was allowed to self-assemble into nanocarriers. The carriers delivered both DOX and siVEGF to human cervical carcinoma cell line HeLa cells and human breast cancer cell line MCF-7 cells.

Zhang. et al. utilized a lipid/calcium/phosphate nanoparticle to encapsulate VEGF siRNA and gemcitabine monophosphate (GMP) for delivery to NSCLC [86]. The membrane/core type nanoparticle consists of a solid calcium phosphate precipitate core, favoring encapsulation of water soluble siRNA and GMP, surrounded by a single lipid bilayer for desirable *in vivo* delivery characteristics. The combination particle successfully down regulated VEGF expression and triggered apoptotic cells in the tumor (~30%), significant when compared with the 12% triggered by the same vehicle carrying GMP alone.

In another study, a “Triple-Punch” nanocarrier of PTX, indocyanine green ICG and sIRNA against the survivin gene were synthesized to destroy MDA-MB-231 cells [87]. The authors synthesized carriers with a lower critical solution temperature (LCST) slightly above body temperature by altering the ratio of poly (2-(2-methoxyethoxy)ethyl methacrylate) polyoligo(ethylene glycol) methacrylate(OEGMA) to OEGMA in the nanocarrier. Local heating by NIR-laser irradiation induced the collapse of micelles to improve delivery effectiveness. This method allowed significantly less of each therapeutic agent to be used for effective antitumor activity (final concentrations: ICG, 0.1 ug/mL; PTX 0.1 ug/mL; survivin siRNA, 100 nM). The 50% inhibitive concentration of free PTX was 100 times more than the concentration of encapsulated drug. Western blot analysis confirmed that expression of survivin protein was down-regulated by carriers containing the siRNA, as compared with the null carriers.

Active targeting is a strategy employed to decrease toxicity of drug delivery *in vivo*. Coupling a cell penetrating peptide (CPP) to the delivery vehicle can increase cellular internalization of the vehicle. Authors in one study were able to co-deliver anti-VEGF shRNA and DOX via denigraft poly-L-lysine (DGL) couple to CPP [88]. Further, CPP was coupled to a pH-sensitive masking peptide by a matrix metalloproteinase 2 (MMP2) substrate. The masking peptide loses its effectiveness in the low pH tumor microenvironment, followed by MMP2 substrate cleavage. Cell internalization is then enhanced by the exposed CPP. Enhanced uptake was observed as well as significant improvement in overall survival of tumor-bearing mice.

## Conclusion and Future Studies

In summary, this review shows that multiple combinations of different types of drugs ranging from chemotherapy to photosensitizers to biologics to immunotherapeutics and gene therapies have been successfully combined into targeted and non-targeted nanoparticles like liposomes, polymeric nanoparticles, micelles, nanostars and nanodiamonds towards the goal of significantly enhancing treatment efficacies while reducing morbidity in preclinical models of cancer. Multifunctional nanomedicines for intelligently designed, mechanism-based combination therapies are likely to deliver the right drugs to the right place at the right time for optimal treatment responses with reduced morbidity fulfilling the promise of nanotechnology for medical applications. Theranostics, the combination of multi-drug therapy and diagnostics may be the next big step in this continued fight against cancer.

With promise comes caution. No nanomedicine that combines two or more drugs in a single platform has been approved for clinical use yet. Some of the designs of the nanoplatforms used for these combinations are fairly complex thereby limiting excitement in the context of successful clinical development. Most of the designs and synthesis procedure outlined provide little to no ratiometric control of the two or more drugs that are trapped inside these nanoparticles. Clearly, more optimization is needed for such nano-combinations to find maximal therapeutic responses. Next generation of nano-combinations need to be easy to manufacture and provide excellent control on drug ratios with exceptional batch-to-batch reproducibility. Another factor rarely considered is, if some of these nanoplatforms are successful in getting clinical approval, what will be the cost of these nano-combinations? The costs of these nanocombinations are likely to be significantly higher than the collective cost of the individual drugs combined. All of these challenges need to be addressed as soon as possible using a multidisciplinary approach with collaborations between academia, the pharmaceutical industry and the regulatory bodies involved to ensure that nano-combination therapy delivers on its promise of better treatment outcomes while severely reducing morbidity thus improving the quality of life in cancer patients.

## Figures and Tables

**Table 1:** FDA approved combination chemotherapy.

Drug 1	Drug 2	Type of Cancer	Date
Lenvatinib capsule (cytotoxic)	Everolimus (cytotoxic)	Advanced renal cell carcinoma	May 2016
Obinutuzumab (Biologic)	Bendamustine (cytotoxic)	Follicular Lymphoma After use of obinutuzumab monotherapy	February 2016
Palbociclib (cytotoxic)	Fulvestrant (Cytotoxic)	HR+, HER2-advanced or metastatic breast cancer	February 2016
Elotuzumab (Biologic)	Lenalidomide, and Dexamethasone (Cytotoxic)	Multiple melanoma	November 2015
Necitumumab (Biologic)	Gemcitabine, and cisplatin (Cytotoxic)	Metastatic squamous non-small cell lung cancer	November 2015
Trametinib (Cytotoxic)	Dabrafenib (Cytotoxic)	Unresectable or metastatic melanoma with BRAF V600E or V600K mutations	November 2015
Ixazomib (Cytotoxic)	Lenalidomide and Dexamethasone (Cytotoxic)	Multiple myeloma	November 2015
Irinotecan liposome (Cytotoxic)	Fluorocil and Leucovorin (Cytotoxic)	Metastatic adenocarcinoma of pancreas	October 2015
Nivolumab (Biologic)	Ipilimumab (Biologic)	BRAF V600 wild-type, unresectable or metastatic melanoma	September 2015
Carfilzomib (Cytotoxic)	Lenalidomide and Dexamethasone (Cytotoxic)	Relapsed multiple myeloma	July 2015
Ramucirumab (Biologics)	FOLFIRI (Cytotoxic)	Metastatic colorectal cancer	April 2015
Dinutuximab (Biologics)	Garnulocyte-macrophage colony stimulating factor, interleukin-2, 13 cis-retinoic acid (Biologic)	Neuroblastoma in pediatric patients	March 2015
Panobinostat (Cytotoxic)	Bortezomib and Dexamethasone (Cytotoxic)	Multiple myeloma	February 2015
Palbociclib (Cytotoxic)	Letrozole (Cytotoxic)	Postmenopausal women with ER+HER2-advanced breast cancer.	February 2015
Ramucirumab (Biologic)	Docetaxel (Cytotoxic)	Non-small cell lung cancer.	December 2014
Bevacizumab solution (Biologic)	Paclitaxel, or Topotecan (Cytotoxic)	Platinum resistant, recurrent epithelial ovarian, fallopian tube, or primary peritoneal cancer.	November 2014
Ramucirumab (Biologic)	Paclitaxel (Cytotoxic)	Advanced gastric or gastroesophageal junction (GEJ) adenocarcinoma	November 2014
Idelalisib (Cytotoxic)	Rituximab (Biologic)	Relapsed Chronic Lymphocytic Leukemia (CLL)	July 2014
Ofatumumab (Biologic)	Chlorambucil (Cytotoxic)	Previously untreated patients with CLL	April 2014
Trametinib (Cytotoxic)	Dafrafenib (Cytotoxic)	Metastatic Melanoma with a BRAF V600E or V600K mutation	January 2014
Obinutuzumab (Biologic)	Chlorambucil (Cytotoxic)	Previously untreated CLL	November 2013
Pertuzumab (Biologic)	Trastuzumab (Biologic) / Docetaxel (Cytotoxic)	Neoadjuvant treatment of patients with HER2-positive breast cancer.	September 2013
Abraxane (Cytotoxic)	Gemcitabine (Cytotoxic)	Metastatic adenocarcinoma of the pancreas.	September 2013
Bevacizumab (Biologic)	fluoropyrimidine-irinotecan or fluoropyrimidine-oxaliplatin (Cytotoxic)	Metastatic colorectal cancer (mCRC)	January 2013
